# Isolated ascending aorta dilatation is associated with increased risk of abdominal aortic aneurysm

**DOI:** 10.1186/s13019-021-01488-w

**Published:** 2021-04-23

**Authors:** Enrique Gallego-Colon, Chaim Yosefy, Evgenia Cherniavsky, Azriel Osherov, Vladimir Khalameizer, Xavier Piltz, Marina Pery, Sharon Bruoha, Jamal Jafari

**Affiliations:** 1 Cardiology Department, Barzilai Medical Center Campus, Barzilai University Medical Center, Ben-Gurion University, Ashkelon, Israel; 2 Department of Medical Imaging, Barzilai University Medical Center, Ben-Gurion University, Ashkelon, Israel

**Keywords:** Abdominal aortic aneurysm, Aortic dilatation, Screening, Computed tomography, Transthoracic echocardiography

## Abstract

**Background:**

Abdominal aortic aneurysm (AAA) is an asymptomatic condition characterized by progressive dilatation of the aorta. The purpose of this study is to identify important 2D-TTE aortic indices associated with AAA as predictive tools for undiagnosed AAA.

**Methods:**

In this retrospective study, we evaluated the size of the ascending aorta in patients without known valvular diseases or hemodynamic compromise as predictive tool for undiagnosed AAA. We studied the tubular ascending aorta of 170 patients by 2-dimensional transthoracic echocardiography (2D-TTE). Patients were further divided into two groups, 70 patients with AAA and 100 patients without AAA with normal imaging results.

**Results:**

Dilatation of tubular ascending aorta was measured in patients with AAA compared to the group with absent AAA (37.5 ± 4.8 mm vs. 31.2 ± 3.6 mm, *p* **<** 0.001, respectively) and confirmed by computed tomographic (CT) (35.6 ± 5.1 mm vs. 30.8 ± 3.7 mm, *p* **<** 0.001, respectively). An increase in tubular ascending aorta size was associated with the presence of AAA by both 2D-TTE and CT (*r* = 0.40, *p* **<** 0.001 and *r* = 0.37, *p* **<** 0.001, respectively). The tubular ascending aorta (D diameter) size of ≥33 mm or ≥ 19 mm/m^2^ presented with 2–4 times more risk of AAA presence (OR 4.68, CI 2.18–10.25, *p* = 0.001 or OR 2.63, CI 1.21–5.62, *p* = 0.02, respectively). In addition, multiple logistic regression analysis identified tubular ascending aorta (OR 1.46, *p* < 0.001), age (OR 1.09, *p* = 0.013), gender (OR 0.12, *p* = 0.002), and LVESD (OR 1.24, *p* = 0.009) as independent risk factors of AAA presence.

**Conclusions:**

An increased tubular ascending aortic diameter, measured by 2D-TTE, is associated with the presence of AAA. Routine 2D-TTE screening for silent AAA by means of ascending aorta analysis, may appear useful especially in older patients with a dilated tubular ascending aorta (≥33 mm).

**Supplementary Information:**

The online version contains supplementary material available at 10.1186/s13019-021-01488-w.

## Background

Abdominal aortic aneurysm (AAA) is a local dilatation of the abdominal aorta exceeding the normal diameter by 50%, or a dilatation of more ≥30 mm in size [1]. Commonly diagnosed in the fifth decade of life, AAA displays slow and variable rate of progression or remains stable for several years. The AAA prevalence can range from 1.3% in men aged 55–64, 9.1% in patients between 65 and 74 and 16.8% in patients between 75 and 84, and 22.0% in patients ≥85 year [2]. Smoking, hypertension, elevated cholesterol levels, and obesity are among the most common modifiable risk factors [3–5]. The current U.S. Preventive Services Task Force (USPSTF) recommendation statement on screening for abdominal aortic aneurysm, recommends 1-time screening for AAA with ultrasonography in men aged 65 to 75 years who have ever smoked (B recommendation) [6]. However, a recent study by Carnevale and collaborators emphasized the need of expanding the established USPSTF screening guidelines to include the expanded Society for Vascular Surgery (SVS) criteria which may potentially double the number of patients identified with AAA [7]. Since abdominal aortic dilatation is usually asymptomatic, and screening programs are not widely implemented, aneurysm of the aorta is usually discovered incidentally or upon rupture. In those patients suffering from a ruptured abdominal aortic aneurysm, the mortality rate is between 60 and 80%, with an additional elevated operative mortality [8–11].

In this study, we evaluated the utility of the ascending aorta examination by 2D-TTE for AAA screening. In addition, we aimed to identify important 2D-TTE aortic indices associated with AAA as predictive tools for undiagnosed AAA. The identification of patients with altered aortic indices that can raise the suspicion of AAA during routine 2D-TTE could prompt immediate AAA evaluation.

## Methods

### Cohort description

For this retrospective study, we analyzed detailed patient data from 2012 to 2019 retrieved from a picture archiving computer system (PACS) database. Patients diagnosed with AAA by computed tomographic (CT) imaging that also underwent 2D-TTE were selected for this study. Aneurysm of the abdominal aorta was defined as an abdominal aorta diameter of ≥3 cm or an aortic diameter 1.5 times larger than the adjacent segment based on current guidelines [11]. The study population included 170 patients divided into two groups, 70 patients with diagnosed AAA and 100 patients without AAA (absent AAA group) with normal imaging results. The exclusion criteria included patients with dilated ascending aorta due to cardiac abnormalities, valvular disease (e.g.: aortic stenosis or regurgitation), post-stenotic dilatation, mechanical or infected valves, aortic dissection and connective tissue disorders (e.g.: Marfan syndrome, Ehlers-Danlos syndrome, or bicuspid valve). Diabetes mellitus was defined as HbA1c ≥ 6.5%, or fasting plasma glucose level ≥ 126 mg/dL in three separate measurements. The study protocol adhered to the Declaration of Helsinki and was approved by the institutional review board of Barzilai Medical Center (BRZ-0090-20).

### 2-dimensional transthoracic echocardiography

**Fig. 1:**
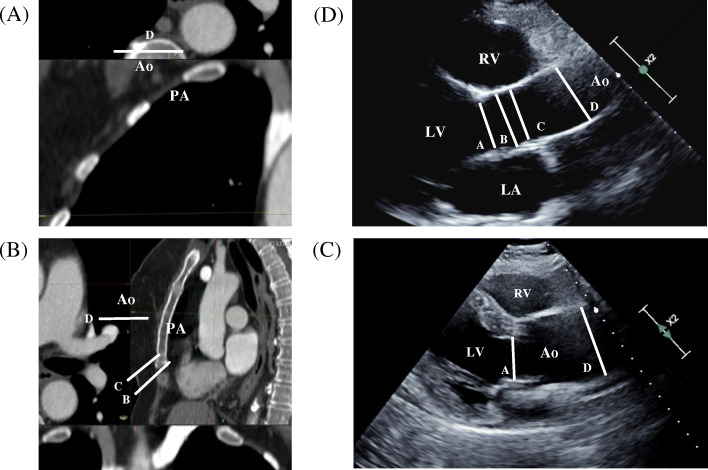
Cardiovascular imaging of the ascending aorta by (**a** and **b**) computed-tomography and by (**c** and **d**) 2D-transthoracic echocardiography. Aortic annulus (D diameter). Sinuses of Valsalva (B diameter). Sinotubular junction (C diameter). Proximal (tubular) ascending aorta (D diameter). PA, pulmonary artery. Ao, Aorta. RV, right ventricle. LV, left ventricle

Routine echocardiographic evaluation was performed by three registered sonographers (C.Y., X.P., and M.P.) according to the recommended guidelines [12, 13]. The thoracic aorta, from the aortic annulus to the innominate artery, was measured at the aortic annulus (A diameter), at the sinus of Valsalva (B diameter), at the sinotubular junction (C diameter), and at the proximal (tubular) ascending aorta (D diameter) (Fig. 1) [13]. The aortic annulus (A diameter) was analyzed to evaluate valvular dilatation as part of the exclusion criteria. Changes in the tubular ascending aorta (D diameter) were measured at the level of the ascending aorta, 3 cm above the aortic valve [13]. The echocardiographic measurements were obtained in standard parasternal long axis views and normalized for the body surface area [13]. The aortic diameter was measured from inner edge-to-inner edge during diastole to increase reproducibility. To reduce overestimation of actual dimensions, the aorta was measured along the axis perpendicular to its long axis, to avoid obtaining an oblique imaging plane. All routine echocardiography exams employed an EPIQ 7 and iE33 echocardiographic machine (Philips Medical Systems, Andover, MA). All images were digitally stored for offline analysis (QLAB 10.0 cardiac 3DQ, Philips Medical Systems).

### Computed-tomographic angiography

CT angiography of the abdominal aorta was performed using a 64-channel MDCT (Brilliance, Philips, Eindhoven, Holland), which covered the region between the thoracic inlet and the common femoral arteries. The thoracic aorta, from the aortic annulus to the innominate artery, was measured at the aortic annulus (A diameter), at the sinus of Valsalva (B diameter), at the sinotubular junction (C diameter), and at the proximal (tubular) ascending aorta (D diameter) (Fig. 1) [13]. An average value for the abdominal aortic diameter was calculated for infra and/or suprarenal aneurysm presentation. The pre-contrast phase was acquired with a collimation of 2.5 mm, 120 kVp, and 320 mAs. The arterial and venous post-contrast phases were both acquired with 0.625 mm slice collimation, a helical pitch of 0.703, a tube rotation velocity of 0.5 per second, tube voltage 120 kVp, and planned tube current-time product 350 mAs; reconstructed to a 1.0-mm slice thickness.

### Statistical analysis

The results are presented as the mean ± standard deviation (SD) for continuous variables with normal distribution, and as number and percentage of total patients for categorical data. T-test or one-way Analysis of Variance (ANOVA) with Bonferroni’s multiple comparison tests was used for comparison of continuous variables. Proportions were compared with contingency tables followed by the chi-square test. Pearson’s statistics were used to assess the relationship between variables. Multiple logistic regression analysis was used to ascertain independent variables associated with AAA. The results are presented as the odds ratio (OR) with a 95% confidence interval (CI). A two-sided *p*-value < 0.05 was considered statistically significant. Statistical analysis was performed with SPSS software version 21.0 statistical package (SPSS IBM. Inc.).

## Results

We analyzed data from 170 patients divided into two groups, the AAA group comprised of 70 patients diagnosed with AAA and, the control group without AAA (No AAA group) comprised of 100 patients (Table 1). No significant differences in age, body mass index, body surface area and heart rate between the two groups were observed (Table 1). Compared to the control group, patients with AAA were older (74.43 ± 12.86 vs. 77.97 ± 9.47, *p* = 0.053) with higher prevalence of hypertension (55% vs. 84%; *p* < 0.001), altered lipid profile (42% vs. 71%; *p* < 0.001), stroke (10% vs. 21%; *p* = 0.05), ischemic heart disease (21% vs. 70%, *p* < 0.001), peripheral vascular disease (3% vs. 24%, *p* < 0.001), and diabetes (16% vs. 30%, *p* = 0.04) (Table 1).

**Table 1:** Clinical characteristics of the cohort

Variable	Abdominal Aortic Aneurysm (AAA)		***p***-value
	Control No AAA (***n*** = 100)	AAA (***n*** = 70)	
**Patient characteristics**			
Age (years), mean ± SD	74.43 ± 12.86	77.97 ± 9.47	0.053
Women, n (%)	52 (52)	10 (14.2)	0.001
BMI, mean ± SD	22.3 ± 3.85	22.16 ± 3.53	0.83
BSA (m^2^), mean ± SD	1.77 ± 0.21	1.83 ± 0.18	0.09
Heart Rate	74.68 ± 17.19	68.15 ± 14.42	0.07
**Comorbidities**			
Hypertension, n (%)	55 (55)	59 (84)	0.001
Dyslipidemia, n (%)	42 (42)	50 (71)	0.001
Stroke, n (%)	10 (10)	15 (21)	0.05
Ischemic heart disease, n (%)	21 (21)	49 (70)	0.001
Peripheral vascular diseases, n (%)	3 (3)	17 (24)	0.001
Current smoker, n (%)	15 (15)	15 (21)	0.31
Diabetes mellitus, n (%)	16 (16)	21 (30)	0.04

*BMI* Body mass index. *BSA* Body surface area

**Fig. 2:**
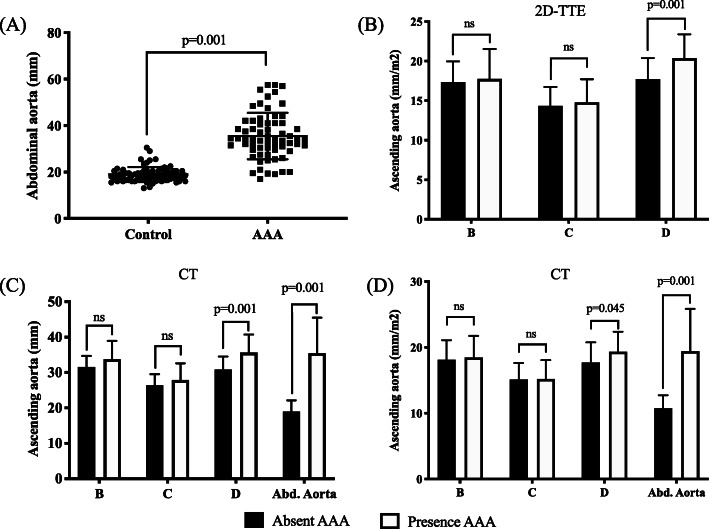
Analysis of tubular ascending aorta diameters in patients with presence of abdominal aortic aneurysm (AAA) and absence AAA. **a** Abdominal aortic diameter in presence (AAA group) and absence (Control group) of AAA. Difference in ascending aorta diameters by (**b**) 2D-transthoracic echocardiography and (**c** and **d**) CT scan in the absence (black) and presence (white) of AAA. Sinuses of Valsalva (B diameter). Sinotubular junction (C diameter). Abd. aorta, abdominal aorta

**Fig. 3:**
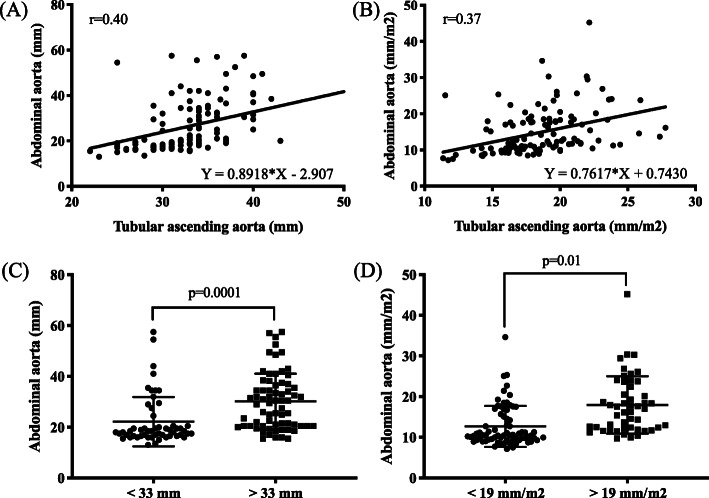
Association between abdominal aortic size and proximal (tubular) ascending aorta. **a** and **b** Pearson correlation coefficient of the tubular ascending aorta (D diameter), mm (*r* = 0.40, *p* < 0.001) and mm/m^2^ (*r* = 0.37, *p* < 0.001), by CT. Abdominal aortic diameter threshold for the D measurement, (**c**) mm and (**d**) mm/m^2^, by 2D-transthoracic echocardiography

Clinical and 2D-TTE measurements in patients with or without AAA are shown in Table 2. No significant differences were observed in left ventricular end diastolic diameter, intraventricular septal thickness, right ventricular end diastolic diameter, left atrium (LA)-AP diameter, LA area, right atrium (RA), E/E’ and E/A ratio between the two groups. Conversely, left ventricular end systolic diameter (29.4 ± 4.4 mm vs. 37.5 ± 9.4 mm; *p* = 0.005, Table 2) and left ventricular ejection fraction (60.4 ± 3.9% vs. 50.0 ± 6.5%; *p* < 0.001) were significantly higher in the AAA group than in the control group (No AAA group) (Table 2). No significant differences in ascending aorta values were observed when both 2D-TTE and CT imaging studies were compared (Figure Sup. 1). Abdominal aortic values revealed an average aneurysm of ≥30 mm in the AAA group when compared to the control group (18.9 ± 3.2 mm vs. 35.5 ± 10 mm, *p* < 0.001; Fig. 2a and Table 2). Interestingly, 2D-TTE analyses of the ascending aorta showed significant differences between patients with and without AAA. Compared to the control group, the 2D-TTE analyses of the ascending aorta indicated a dilatation at the level of the tubular ascending aorta (D diameter) in patients diagnosed with AAA (31.2 ± 3.6 mm vs. 37.5 ± 4.8 mm; *p* < 0.001 or 17.7 ± 2.7 mm/m^2^ vs. 20.4 ± 3.0 mm/m^2^; *p* < 0.001) (Fig. 2b and Table 2). Subsequently, CT analysis of the tubular ascending aorta confirmed the dilatation in D diameter for the AAA group as observed by 2D-TTE imaging (30.8 ± 3.7 mm vs. 35.6 ± 5.1 mm; *p* < 0.001 and 17.7 ± 3.0 mm/m^2^ vs. 19.3 ± 3.0 mm/m^2^; *p* = 0.045) (Fig. 2c, d and Table 2). In the AAA group, the Pearson’s correlation coefficient indicated that higher D measurement values correlated significantly with the abdominal aortic size (*r* = 0.40, *p* < 0.001 and *r* = 0.37, *p* < 0.001, respectively; Fig. 3a and b). In addition, we observed 2–4 times more risk of finding abdominal aortic aneurysm by 2D-TTE in patients with a D value of ≥33 mm (OR 4.68, CI 2.18–10.25, *p* < 0.001, Fig. 3c) or an index ≥19 mm/m2 (OR 2.63, CI 1.21–5.62, *p* = 0.02, Fig. 3d). Multiple logistic regression analysis revealed that age (OR 1.09, CI 1.03–1.17, *p* = 0.013), gender (OR 0.12, CI 0.30–0.47, *p* = 0.002), tubular ascending aorta size (D diameter, OR 1.46, CI 1.18–1.82, *p* < 0.001), and LVESD (OR 1.24, CI 1.05–1.45, *p* = 0.009) were the independent predictors of AAA (Table 3).

**Table 2:** Comparison of echocardiographic characteristics

Variable	Abdominal Aortic Aneurysm		***p***-value
	Control No AAA (***n*** = 100)	AAA (***n*** = 70)	
**Standard echocardiographic measurements**			
LVEDD (mm), mean ± SD	45.6 ± 4.7	52.0 ± 7.0	0.07
LVESD (mm), mean ± SD	29.4 ± 4.4	37.5 ± 9.4	0.005
IVS (mm), mean ± SD	10.32 ± 1.8	11.6 ± 1.7	0.99
LVEF (%), mean ± SD	60.4 ± 3.9	50.0 ± 6.5	0.001
RVEDD (mm), mean ± SD	35.8 ± 5.1	40.3 ± 6.5	0.51
LA-AP (mm), mean ± SD	36.5 ± 6.8	40.2 ± 7.1	0.72
LA area (mm), mean ± SD	20 ± 5.5	24.5 ± 7	0.51
RA area (mm), mean ± SD	16.1 ± 5.1	19.3 ± 6.8	0.86
E/E’	9.1 ± 4.1	11.2 ± 4.1	0.99
E/A ratio	1 ± 0.4	0.9 ± 0.6	0.99
**Ascending aorta measurements by 2D-TTE**			
Aortic annulus, A, (mm)	19.8 ± 1.6	20.8 ± 1.4	0.67
Aortic annulus, A, (mm/m2)	11.3 ± 1.3	11.4 ± 1.1	0.99
Sinus of Valsalva, B, (mm)	30.6 ± 4.3	32.7 ± 6.2	0.051
Sinus of Valsalva, B, (mm/m2)	17.3 ± 2.6	17.8 ± 3.8	0.72
Sinotubular junction, C, (mm)	25.3 ± 4.5	27.3 ± 4.9	0.08
Sinotubular junction, C, (mm/m2)	14.3 ± 2.4	14.8 ± 2.9	0.64
Tubular ascending aorta, D, (mm)	31.2 ± 3.6	37.5 ± 4.8	0.001
Tubular ascending aorta, D, (mm/m2)	17.7 ± 2.7	20.4 ± 3.0	0.001
**Ascending and abdominal aorta measurements by CT**			
Sinus of Valsalva, B, (mm)	31.5 ± 3.1	33.8 ± 5.2	0.07
Sinus of Valsalva, B, (mm/m2)	18.1 ± 2.9	18.5 ± 3.3	0.96
Sinotubular junction, C, (mm)	26.4 ± 3.2	27.9 ± 4.7	0.37
Sinotubular junction, C, (mm/m2)	15.1 ± 2.5	15.2 ± 2.9	0.99
Tubular ascending aorta, D, (mm)	30.8 ± 3.7	35.6 ± 5.1	0.001
Tubular ascending aorta, D, (mm/m2)	17.7 ± 3.0	19.3 ± 3.1	0.045
Abdominal aorta (mm)	18.9 ± 3.2	35.5 ± 10	0.001
Abdominal aorta (mm/m2)	10.7 ± 2	19.4 ± 6.4	0.0001

*LVEDD* Left ventricle end diastolic diameter. *LVESD* Left ventricle end systolic diameter. *IVS* Interventricular septum. *LVEF* Left ventricle ejection fraction. *RVEDD* Right ventricle end diastolic diameter. *LA-AP* Left atrial anterior-posterior diameter. *LA Area* Left atrial area. *RA Area* Right atrial area. *E/A* Early to late mitral flow. *CT* Computed tomography scan. *2D-TTE* 2-Dimensional transthoracic echocardiography

**Table 3:** Multiple logistic regression analysis showing independent predictors of abdominal aortic aneurism

Variables	***p***-value	OR	95% CI	
			Lower	Upper
Age (years)	0.006	1.09	1.03	1.17
Gender, n (%)	0.002	0.12	0.30	0.47
Tubular ascending aorta (mm)	0.001	1.46	1.18	1.82
LVESD (mm)	0.009	1.24	1.05	1.45
LVEF (%)	0.82	0.98	0.87	1.12

*OR* Odd ratio. *CI* Confidence interval. *LVESD* Left ventricle end systolic diameter. *LVEF* Left ventricle ejection fraction

## Discussion

Abdominal aortic aneurysm is a progressive condition with an increased risk of aortic dissection and mortality [8, 14]. In this study, our results indicate that 2D-TTE imaging is comparable to CT methodology for measuring and estimating ascending aorta diameters during routine echocardiographic examination. Routine 2D-TTE procedures include, but are not limited to, initial and supplemental tests, evaluation of end organ damage (e.g.: hypertension, diabetes mellitus), evaluation of cardiac and aortic structure and function (e.g.: left atrial appendage, left ventricular hypertrophy and diastolic dysfunction, prosthetic heart valves, paravalvular abscesses, patients on ventilators, or with chest wall injuries), intraoperative TTE, guidance of transcatheter procedures (e.g.: septal defect closure, or atrial appendage obliteration, transcatheter valve procedures), and critically ill patients [15]. In addition, we show that patients aged over 75, primarily non-smokers, without known valvular diseases or hemodynamic compromise, but with increased proximal (tubular) ascending aorta (D diameter) during routine echocardiographic measurements, may present with silent AAA. In fact, an increased proximal (tubular) ascending aorta (D diameter) represents an independent predictor of a silent AAA with a threshold of ≥33 mm or ≥ 19 mm/m^2^. We hypothesized that in larger prospective studies, the B diameter can also become significantly dilated. Conversely, the fibrous portion of the A and C diameters, can mask progression of the dilatation in those areas.

The abdominal aorta can be relatively easily visualized to the left of the inferior vena cava in sagittal (superior–inferior) subcostal views [16]. Although, 2D-TTE transducers are not optimal for aneurysm detection, detection of an abnormal abdominal aorta can prompt further imaging studies to confirm the presence of AAA. Upon finding of abnormal ascending aorta indices, we recommend screening of the abdominal aorta by means of 2D-TTE, as show to be feasible with minimal additional time and cost compared to separate abdominal ultrasound examination [16, 17]. Our recommendation extends the current guidelines which recommend 1-time screening for AAA with ultrasonography in men aged 65 to 75 years who have ever smoked [6].

Interestingly, patients with diabetes may have a lower incidence of abdominal aortic aneurysm, although the link between diabetes and AAA development and expansion is unclear [18, 19]. In our study, we observed a significant number of diabetic patients in the AAA group compared to the control group (no AAA group). This seemingly opposing results can be explained by the fact we did not include within the aims of the study the evaluation of diabetes, hence, patient stratification was not addressed towards that end. To conclude, routine 2D-TTE examination of the ascending aorta is a rapid, accurate and cost-effective tool to identify a ‘silent’ high-risk AAA population for which further evaluation may be beneficial [17, 20]. These are particularly relevant when screening for AAA may be overlooked, or screening programs with ultrasonography may not be fully implemented [6, 21]. Clinical awareness, and performance of comprehensive echocardiographic analysis, can help in early diagnosis to reduce AAA-associated risks, reduce mortality and morbidity as well as the economic burden. The limitations of the present study are its non-randomized, retrospective observational design and the limited number of patients. Selection bias is a limitation of the studies included in the analysis. A larger prospective study must be conducted to monitor and determine the presence of AAA in patients with isolated dilated ascending aorta without any associated cardiovascular complications.

## Conclusions

In our study, we observed that ascending aorta indices obtained during routine 2D-TTE in asymptomatic patients, without known valvular diseases or hemodynamic compromise, can indicate the presence silent AAA. Hence, we recommend a review of current recommendations and expand AAA screening to routine 2D-TTE.

## Supplementary Information

**Additional file 1.**

## Data Availability

The datasets used and/or analysed during the current study are available from the corresponding author on reasonable request.

## Abbreviations

2D-TTE: 2-Dimensional transthoracic echocardiography
AAA: Abdominal aortic aneurysm
ANOVA: Analysis of variance
BMI: Body mass index
BSA: Body surface area
CI: Confidence interval
CT: Computed tomographic
E/A: Early to late mitral flow
IVS: Interventricular septum
LA Area: Left atrial area
LA-AP: Left atrium anterior-posterior diameter
LA: Left atrium
LVEDD: Left ventricle end diastolic diameter
LVEF: Left ventricle ejection fraction
LVESD: Left ventricle end systolic diameter
OR: Odd ratio
PACS: Picture archiving computer system
RA Area: Right atrial area
RVEDD: Right ventricle end diastolic diameter
SD: Standard deviation
SVS: Society for vascular surgery
USPSTF: The current U.S. preventive services task force
