# Correction to: Isolated ascending aorta dilatation is associated with increased risk of abdominal aortic aneurysm

**DOI:** 10.1186/s13019-021-01553-4

**Published:** 2021-06-09

**Authors:** Enrique Gallego-Colon, Chaim Yosefy, Evgenia Cherniavsky, Azriel Osherov, Vladimir Khalameizer, Xavier Piltz, Marina Pery, Sharon Bruoha, Jamal Jafari

**Affiliations:** 1 Cardiology Department, Barzilai Medical Center Campus, Barzilai University Medical Center, Ben-Gurion University, Ashkelon, Israel; 2 Department of Medical Imaging, Barzilai University Medical Center, Ben-Gurion University, Ashkelon, Israel

**Correction to: J Cardiothorac Surg 16, 108 (2021)**

**https://doi.org/10.1186/s13019-021-01488-w**

The original article [1] contained typesetting errors in Fig. 1 which have since been corrected.
