# Supplementary material for: Isolated ascending aorta dilatation is associated with increased risk of abdominal aortic aneurysm
Source: J Cardiothorac Surg. 2021 Apr 23;16:108. doi: 10.1186/s13019-021-01488-w (PMC8063334; doi:10.1186/s13019-021-01488-w)
Supplement: Supplementary file 1 — Additional file 1. [file 13019_2021_1488_MOESM1_ESM.pdf]

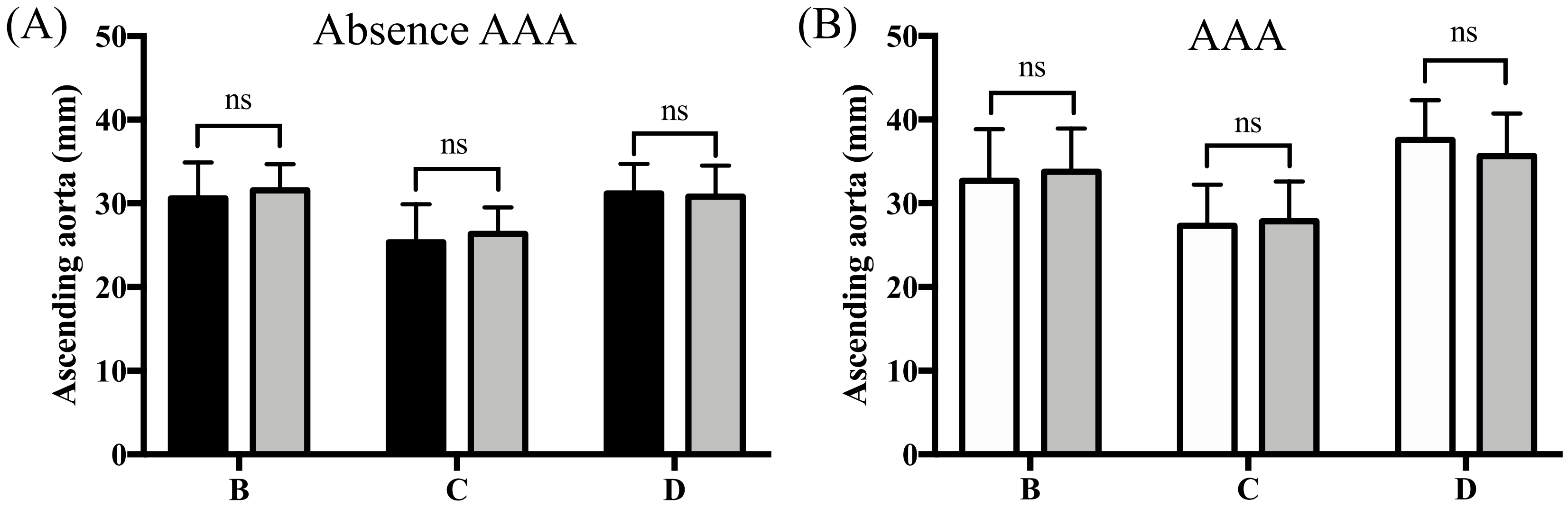

Supplementary figure 1. Ascending aorta diameters in (A) absence AAA and (B) in AAA patients by 2D-TTE and CT. Black bars, 2D-TTE measurement. Gray bars, CT measurement. White bars, AAA by 2D-TTE. Sinuses of Valsalva (B diameter). Sinotubular junction (C diameter). Proximal (tubular) ascending aorta (D diameter).
